# NBOMes–Highly Potent and Toxic Alternatives of LSD

**DOI:** 10.3389/fnins.2020.00078

**Published:** 2020-02-26

**Authors:** Jolanta B. Zawilska, Monika Kacela, Piotr Adamowicz

**Affiliations:** ^1^Department of Pharmacodynamics, Medical University of Łódź, Łódź, Poland; ^2^Department of Forensic Toxicology, Institute of Forensic Research, Kraków, Poland

**Keywords:** new psychoactive substances, NBOMe, phenethylamines, psychedelics, toxicity, metabolism, analytical methods

## Abstract

Recently, a new class of psychedelic compounds named NBOMe (or 25X-NBOMe) has appeared on the illegal drug market. NBOMes are analogs of the 2C family of phenethylamine drugs, originally synthesized by Alexander Shulgin, that contain a *N*-(2-methoxy)benzyl substituent. The most frequently reported drugs from this group are 25I-NBOMe, 25B-NBOMe, and 25C-NBOMe. NBOMe compounds are ultrapotent and highly efficacious agonists of serotonin 5-HT_2A_ and 5-HT_2C_ receptors (Ki values in low nanomolar range) with more than 1000-fold selectivity for 5-HT_2A_ compared with 5-HT_1A_. They display higher affinity for 5-HT_2A_ receptors than their 2C counterparts and have markedly lower affinity, potency, and efficacy at the 5-HT_2B_ receptor compared to 5-HT_2A_ or 5-HT_2C_. The drugs are sold as blotter papers, or in powder, liquid, or tablet form, and they are administered sublingually/buccally, intravenously, via nasal insufflations, or by smoking. Since their introduction in the early 2010s, numerous reports have been published on clinical intoxications and fatalities resulting from the consumption of NBOMe compounds. Commonly observed adverse effects include visual and auditory hallucinations, confusion, anxiety, panic and fear, agitation, uncontrollable violent behavior, seizures, excited delirium, and sympathomimetic signs such mydriasis, tachycardia, hypertension, hyperthermia, and diaphoresis. Rhabdomyolysis, disseminated intravascular coagulation, hypoglycemia, metabolic acidosis, and multiorgan failure were also reported. This survey provides an updated overview of the pharmacological properties, pattern of use, metabolism, and desired effects associated with NBOMe use. Special emphasis is given to cases of non-fatal and lethal intoxication involving these compounds. As the analysis of NBOMes in biological materials can be challenging even for laboratories applying modern sensitive techniques, this paper also presents the analytical methods most commonly used for detection and identification of NBOMes and their metabolites.

## Introducttion

**FIGURE 1:**
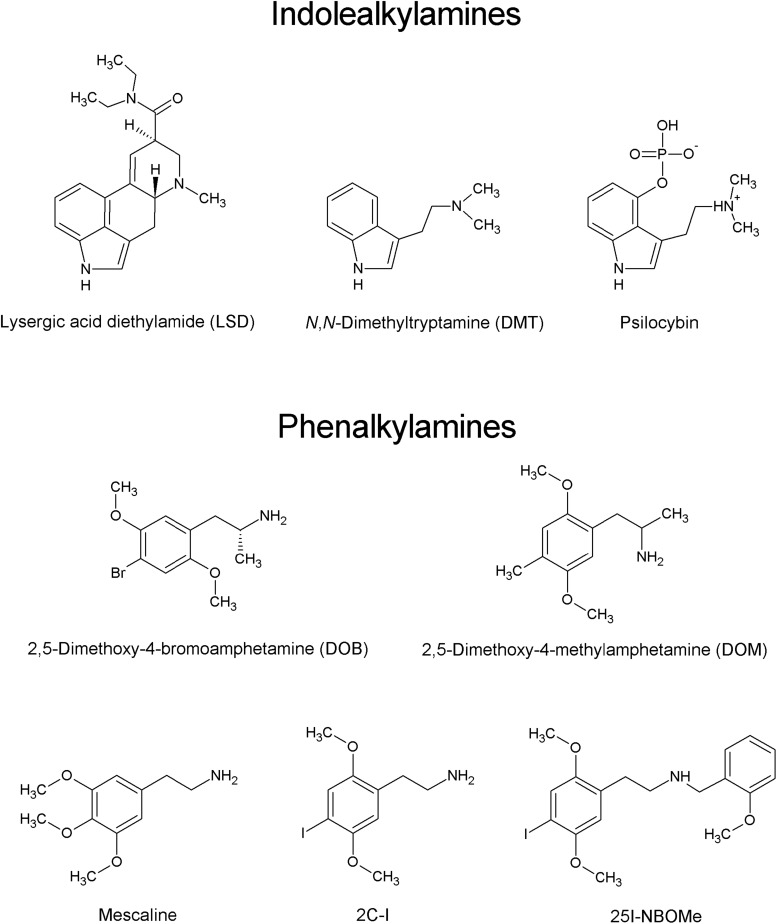
Chemical structures of main classes of psychedelics.

The last decade witnessed the emergence of new psychoactive substances (NPSs), followed by a rapid increase in their prevalence and the constant introduction of new compounds into the clandestine market in order to circumvent the existing laws. From 2009 to 2018, 899 different NPSs were reported worldwide (United Nations Office on Drugs and Crime [UNODC], 2019). Over the course of 2018, a total of 687 NPSs were notified to the European Monitoring Centre for Drugs and Drug Addiction (EMCDDA). In 2018, one new NPS was reported to EMCDDA every week (EMCDDA, 2019). The five main classes of NPSs are synthetic cannabinomimetics, stimulants (dominated by derivatives of cathinone), opioids, psychedelics, and non-pharmaceutical benzodiazepines. By analogy to other NPSs, psychedelic compounds, which produce marked alterations of perception, mood, and cognition, are widely used for recreational purposes. Psychedelics (also called classical or serotoninergic hallucinogens) are divided into two main groups based on their chemical structure: indoleamines (termed also indolealkylamines; e.g., ergolines, including LSD and its analogs, and simple tryptamines, such as *N*,*N*-dimethyltryptamine and 5-methoxy-*N*,*N*-dimethyltryptamine) and phenylalkylamines. Phenylalkylamines are highly selective for serotonin 5-HT_2_ receptors, while indoleamines are relatively non-selective for 5-HT receptors, displaying moderate to high affinity for 5-HT_1_ and 5-HT_2_ receptor subtypes. The phenylalkylamines can be further divided into two subgroups, one group being the phenylisopropylamines (analogs of amphetamine), e.g., 2,5-dimethoxy-4-bromoamphetamine (DOB) and 2,5-dimethoxy-4-methylamphetamine (DOM), and the other being the phenethylamines, including mescaline, 2C-X compounds and their derivatives (Figure 1) (reviewed by Halberstadt, 2017). The name “2C” refers to an acronym created by the ‘godfather’ of psychedelic drugs Alexander Shulgin to describe their chemical structure, where two carbon atoms separate the amine group from the phenyl ring (Shulgin and Shulgin, 1991). The prototype of the 2C series, 2C-B, was synthesized by Shulgin in 1974. Since 2010, a new group of 2C compounds containing an *N*-(2-methoxy)benzyl (*N*-benzoylmethoxy) substituent, known as *N*-(2-methoxybenzyl)phenethylamines (aka 25X-NBOMes or simply NBOMes), has emerged in the illicit drug market. Structure-activity studies indicate that this substituent significantly increases the affinity of the drug toward the 5-HT_2A_ receptor and its pharmacological activity (Hansen et al., 2014). It is important to note that stimulation of the 5-HT_2A_ receptors is required for the psychedelic effects of compounds such as LSD, mescaline, and psilocybin (Glennon et al., 1984; Titeler et al., 1988; Sadzot et al., 1989; Vollenweider et al., 1998; Rickli et al., 2016). The first NBOMes were originally synthesized by Ralf Heim at the Free University of Berlin in a search for pharmacological tools to study the 5-HT_2A_ receptor (Heim, 2003). Since then, [^11^C]25I-NBOMe and [^11^C]25C-NBOMe have been used to map the distribution of 5-HT_2A_ receptors in the brain by positron emission tomography (PET) imaging (Ettrup et al., 2010, 2014; for an excellent review see Poulie et al., 2019).

The first recreationally used drug from this group was 25I-NBOMe (2-(4-iodo-2,5-dimethoxyphenyl)-*N*-[(2-methoxyphenyl)methyl]ethanamine), identified in seven green blotters seized by the Swedish police in May 2012 (EMCDDA, 2014). It is likely that 25I-NBOMe was the first NBOMe to be used recreationally in the United States (Palamar and Le, 2019). Following this, several potent NBOMes were synthesized and introduced into the drug market. In these, the iodine atom was exchanged for other halogens: e.g., bromine (25B-NBOMe) or chlorine (25C-NBOMe), a hydrogen atom (25H-NBOMe), a nitro group (25N-NBOMe) or an organic functional group, - methyl (25D-NBOMe), -ethyl (25E-NBOMe), or –isopropyl (25iP-NBOMe) (Wood et al., 2015; Poulie et al., 2019). Three compounds from this group, namely 25I-NBOMe, 25B-NBOMe, and 25C-NBOMe, accounted for 0.03% of the total quantity of hallucinogens (other than ketamine) seized globally between 2011 and 2017 (United Nations, 2019). In the United States, 2,129 reports for 25I-NBOMe, 1,273 reports for 25C-NBOMe, and 924 reports for 25B-NBOMe were collected by the System to Retrieve Information from Drug Evidence and the National Forensic Laboratory Information System between January 2014 and April 2018 (Drug Enforcement Administration, 2018).

## Methods

This literature review was based on an exhaustive search of PubMed (U.S. National Library of Medicine) that used “NBOMe” and each of the compound names listed in Table 1 as keywords. Only papers written in English and with full texts available by November 2019 were included. Additionally, official reports published by the United Nations Office on Drugs and Crime (UNODOC), EMCDDA, and the World Health Organization (WHO) were studied. Furthermore, in each article and report obtained, references were checked carefully in order to identify possible additional publications missed during the initial search.

**TABLE 1:** Doses and duration of action of NBOMe compounds (TripSit a,b,c,d,e).

Compound (route of administration)	Dose (μg)				Action		
		
	Threshold	Light	Common	Strong	Onset	Duration	After effects
**25B-NBOMe**							
Sublingual	100	100–300	350–500	500–700+	20–40 min	8–12 h	2–6 h
Insufflation	50	50–200	200–350	350–500+	2–5 min	8–12 h	2–6 h
**25C-NBOMe**							
Sublingual	50–250	250–500	500–750	750–1250+	45–90 min	4–10 h	1–24 h
**25E-NBOMe**							
Sublingual	50–100	50–200	200–400	400–800	15–60 min	5–10 h	2–10 h
**25I-NBOMe**							
Sublingual	50–250	200–500	500–750	700–1000+	45–90 min	5–10 h	6–24 h
**25N-NBOMe**							
Buccal/sublingual		100–300	300–800	800–1300+	45–75 min	5–10 h	1–12 h

## Pharmacology of NBOMes

*In vitro* studies indicated that NBOMe compounds are ultrapotent and highly efficacious agonists of 5-HT_2A_ and 5-HT_2C_ receptors (Ki values in low nanomolar range), with more than 1000-fold selectivity for 5-HT_2A_ compared with 5-HT_1A_. The compounds display higher affinity for 5-HT_2A_ receptors than their 2C counterparts and have markedly lower affinity, potency, and efficacy at the 5-HT_2B_ receptor than at 5-HT_2A_ or 5-HT_2C_ (Juncosa et al., 2013; Nichols et al., 2015; Rickli et al., 2015; Elmore et al., 2018; Eshleman et al, 2018). In addition, NBOMes have a significant affinity (Ki < 300 nM) for adrenergic α_1_ receptors but not so H_1_-histamine, dopamine D_1_, D_2_, and D_3_ receptors or the monoamine transporters DAT, NET, or SERT (Nichols et al., 2015; Elmore et al., 2018; Eshleman et al, 2018). Molecular modeling and molecular dynamics simulation studies performed on a human 5-HT_2A_ receptor model identified several amino acid residues as putative binding sites of NBOMes. It is suggested that the binding pocket, localized among transmembranes (TM) III, V, VI, and VII, includes Trp-151(TMIII), Ile-152(TMIII), Asp-155(TMIII), Ser-159(TMIII), Ser-239(TMV), Phe-339 (TMVI), Phe-340(TMVI), Val-336(TMVII), and Tyr-370(TMVII) (Braden et al., 2006; Silva et al., 2011; Ísberg et al., 2011). Among them, a highly conserved Asp-155 forms a salt bridge with the amine nitrogen, Ser-159 and Ser-239 form H-bonds with the 2-methoxy and 5-methoxy group, respectively, and Phe-340 forms a van der Waals interaction with the benzene ring. It should be emphasized that Asp-155, Ser-159, Ser-239, Phe-340 are also important for binding and efficacy of different agonists and partial agonists at 5-HT_2A_ receptor (Silva et al., 2011). On the other hand, the van der Waals interaction between Phe-339 and *N*-benzyl ring of NBOMes and the hydrogen bond formed by Tyr-370 with the 2-position oxygen on this ring are considered to play a key role in the high potency and affinity of these compounds binding to 5-HT_2A_ receptor (Silva et al., 2011; Ísberg et al., 2011).

The activation of cortical 5-HT_2A_ receptors induces the head twitch response (HTR) in mice and rats, also referred to as wet dog shakes (Willins and Meltzer, 1997; Abiero et al., 2019). The HTR is widely used as a behavioral marker for hallucinogen effects in humans (Halberstadt and Geyer, 2018). 25C-NBOMe, 25I-NBOMe, and 25B-NBOMe induced an HTR response in rodents with a potency several-fold higher than their 2C counterparts, 2C-C and 2C-I (Halberstadt and Geyer, 2014; Elmore et al., 2018; Custodio et al., 2019; Herian et al., 2019). Two lines of evidence support a notion that this behavioral effect is mediated by cortical 5-HT_2A_ receptors. Thus, ketanserin, a 5-HT_2A_ antagonist, blocked the 25B-NBOMe-evoked HTR and normalized 5-HT_2A_ mRNA levels in the mouse prefrontal cortex upregulated by a prolonged administration of the drug (Custodio et al., 2019).

Gatch et al. (2017) tested 25B-NBOMe, 25C-NBOMe, and 25I-NBOMe for discriminative stimulus effects similar to a prototypical psychedelic/hallucinogen DOM and to an empathogen, 3,4-methylenedioxymethamphetamine (MDMA). In DOM-trained rats 25B-NBOMe and 25C-NBOMe, but not 25I-NBOMe, fully substituted for this drug. 25B-NBOMe also fully substituted for MDMA. In both tests, the dose-effect curves for 25B-NBOMe had an inverted U-shape. It is suggested that 25B-NBOMe and 25C-NBOMe are most likely used as recreational psychedelics, although 25B-NBOMe may also be used as an empathogenic compound (Gatch et al., 2017). However, the latter assumption should be taken with caution, as some compounds (e.g., fenfluramine) that substitute for MDMA in rats do not produce MDMA-like empathogenic effects in humans (Schechter, 1988).

Using a battery of tests, behavioral effects of 25I-NBOMe (0.5 and 1 mg/kg) were examined in male and female Sprague-Dawley rats (Miliano et al., 2019). In both sexes, the systemic administration of the drug reduced visual object and placing responses–an effect likely related to its pro-hallucinogenic action–and decreased acoustic and tactile responses. Furthermore, by analogy to LSD and MDMA (Halberstadt and Geyer, 2010; Marti et al., 2019), 25I-NBOMe impaired the acoustic startle response [prepulse inhibition, a preclinical behavioral marker of vulnerability to develop a neuropsychiatric disorder (Marti et al., 2019)]. The drug increased body temperature only in females. On the other hand, it exerted an analgesic affect in males. It is suggested that the observed differences could be related to a sex-dependent pharmacodynamic profile of 25I-NBOMe (Miliano et al., 2019).

Psychedelic drugs interact with various neurotransmitter systems, namely serotonergic, glutamatergic, dopaminergic, cholinergic, and GABA-ergic. Among them, the glutamatergic system appears to play a prominent role in the action of these drugs (Aghajanian and Marek, 1999). In vivo microdialysis after systemic administration to rats of DOI, 5-methoxy-*N*,*N*-diisopropyltryptamine (MeO-DIPT) or LSD revealed markedly elevated extracellular levels of glutamate in the cortex (Scruggs et al., 2003; Muschamp et al., 2004; Noworyta-Sokołowska et al., 2016, 2019). DOI also increased the dopamine level in the cortex and ventral tegmental area (VTA) (Bortolozzi et al., 2005; Pehek et al., 2006). Recently, Herian et al. (2019), using microdialysis in freely moving male Wistar-Han rats, demonstrated increased extracellular levels of glutamate, dopamine, and 5-HT in the frontal cortex after administration of 25I-NBOMe. The drug also increased the tissue content of 5-HT and its metabolite hydroxyindoleacetic acid (5-HIAA) but did not affect the tissue content of dopamine and its metabolites: 3,4-dihydroxyphenylacetic acid (DOPAC) and homovanilic acid (HVA). Subsequent studies performed by Miliano et al. (2019) on Spague-Dawley rats, both males and females, showed that 25I-NBOMe increased extracellular dopamine levels in the nucleus accumbens (NAc) shell (but not core) in a sex-independent way. The drug markedly elevated dopamine levels in the medial prefrontal cortex (mPFC) of females but not males. No statistically significant changes in extracellular levels of 5-HT in the three analyzed brain structures were found in both sexes (Miliano et al., 2019). These results suggest that in rats effects of 25I-NBOMe on dopaminergic and serotoninergic transmission depend not only on the brain structure but also strain and sex.

An important question that remains to be fully resolved is whether NBOMe compounds are endowed with an abuse potential. Two behavioral tests, conditioned place preference (CPP) and self-administration (SA), are widely used in studies examining the abuse potential of drugs by analyzing their rewarding and reinforcing effects. 25I-NBOMe (0.3 mg/kg), 25B-NBOMe (1 mg/kg), and 25N-NBOMe (3 mg/kg) produced CPP in mice with a magnitude comparable to 1 mg/kg of methamphetamine (Custodio et al., 2019; Jeon et al., 2019; Seo J.Y. et al., 2019). The 25B-NBOME-elicited CPP was blocked by antagonists of D_1_- and D_2_-dopamine receptors, SCH 23390 and haloperidol, respectively, but was not affected by ketanserin, an observation indicating an important role of dopaminergic transmission in this phenomenon (Custodio et al., 2019). In the SA test performed on mice, 25B-NBOMe used at doses of 0.03, 0.1 and 0.3 mg/kg/infusion significantly increased both a number of infusions/session and an active lever pressing/session, albeit with a weaker potency than methamphetamine (Custodio et al., 2019). On the contrary, 25N-NBOMe (0.01 mg/kg/infusion) weakly increased the number of infusions/session, but not the active lever pressing/session in mice (Seo J.Y. et al., 2019), whereas 25I-NBOMe (0.03 mg/kg/infusion) did not significantly affect these two SA parameters in rats (Jeon et al., 2019). These findings suggest that 25I-NBOMe, 25B-NBOMe, and 25N-NBOMe might have some dependence liability.

As activation of the mesolimbic dopaminergic pathway plays a critical role in drug abuse and addiction, effects of 25N-NBOMe and 25B-NBOMe on the expression of D_1_- and D_2_-dopamine receptors, dopamine transporter (DAT), and tyrosine hydroxylase (TH) at the protein level were examined in two studies. One was performed on mice that, in the course of the CPP test, received in total four injections of 25N-NBOMe (3 mg/kg/injection) and were sacrificed two days after the last injection (Seo J.Y. et al., 2019). In the second study, mice were repeatedly treated with 25B-NBOMe (1 mg/kg) for 7 days; they were sacrificed 30 min after the last injection (Custodio et al., 2019). Results of these studies are, however, not uniform. The level of D_1_ receptor protein was increased in the NAc of mice pretreated with 25B-NBOMe but was not affected in NAc and dorsal striatum (DSt) of 25N-NBOMe mice. Markedly lower levels of D_2_ receptors were found in the ventral tegmental area (VTA) after administration of 25B-NBOMe, in NAc and DSt (25N-NBOMe and 25B-NBOMe). 25N-NBOME decreased expression of DAT and TH in the NAc but not in DSt. 25B-NBOMe-induced a decrease of DAT and did not change TH protein levels in the VTA. Among several factors that might contribute to the above discrepancies, different dosing protocols appears to play an important role.

## Toxicity *In Vitro*

Recent studies demonstrated that NBOMes exhibit neurotoxic and cardiotoxic activity. 25C-NBOMe was cytotoxic against neuronal cell lines SH-SY5Y, PC12, and SN4741 with respective calculated IC_50_ values of 89, 78, and 62 μM. The compound was 56, 25, and 64 times more potent than methamphetamine at reducing the viability of SH-SY5Y, PC12, and SN4741 cells, respectively. The neurotoxic action of 25C-NBOMe involves activation of the MAP/ERK cascade and inhibition of the Akt pathway (Xu et al., 2019). Acute (30 min) and prolonged (5 h) exposure of primary rat cortical cultures to 25B-NBOMe decreased spontaneous neuronal activity, measured as firing rate and burst rate (Zwartsen et al., 2018, 2019). The compound was 10-fold more potent than its precursor, 2C-B. Importantly, neuronal activity did not recover after 19 h of washout following prolonged exposure to 10 and 30 μM of 25B-NBOMe (Zwartsen et al., 2019).

25D-NBOMe and 25C-NBOMe reduced viability of H9c2 cells (cardiomyocytes). Both compounds used at doses of 0.75 and 2 mg/kg downregulated expression levels of p21 (CDC42/RAC)-activated kinase 1 (PAK1), an enzyme with documented cardiac protective effects, and prolonged QT intervals in rat ECG. 25D-NBOMe inhibited the hERG potassium channel, a phenomenon that might play a role in QT interval prolongation (Yoon et al., 2019).

## Available Forms of Products and Pattern of Use

The three most popular compounds from the 25X-NBOMe series are 25I-NBOMe (2-(4-iodo-2,5-dimethoxyphenyl)-*N*-[(2-methoxyphenyl)methyl]ethanamine), 25B-NBOMe (2-(4- bromo-2,5-dimethoxyphenyl)-*N*-[(2-methoxyphenyl)methyl] ethanamine), and 25C-NBOMe (2-(4-chloro-2,5-dimethoxy phenyl)-*N*-[(2-methoxyphenyl)methyl]ethanamine) (Figure 1; Al-Iman and AbdulMajeed, 2017; Halberstadt, 2017; Palamar and Le, 2019). 25I-NBOM is known under street names “Solaris”, “25I”, “Dots”, “legal acid”, “N-Bomb”, “NE-BOME”, “Smiles”, “INBMeO”, “BOM-Cl”, “Hoffman”, “N-boom”, and “Holand Film”; 25C-NBOMe as “C-Boom”, “Cimbi-82”, “Pandora”, and “Dime”; and 25B-NBOMe as “Nova”, “legal acid”, “NBomb”, “NE-BOME”, “New Nexus”, “NBOMe-2-B”, and “BOM 2-CB” (Zuba et al., 2013; EMCDDA, 2014; World Health Organization, 2014a, b; Al-Iman and AbdulMajeed, 2017).

NBOMes are typically available in the form of preloaded paper doses (blotters) with images and logos from popular cartoons and music/movie posters and, less frequently, in powder or liquid form (Zuba et al., 2013; EMCDDA, 2014; World Health Organization, 2014a, b; Andrabi et al., 2015; Halberstadt, 2017). As NBOMes undergo extensive first-pass metabolism (see section BIOTRANSFORMATION), preferred and common patterns of their use include sublingual, buccal, and nasal. Thus, 25C-NBOMe and 25I-NBOMe are usually taken by holding the blotter in the mouth (sublingually or buccally), insufflated as powder, or in solution as a nose spray (Zuba et al., 2013; EMCDDA, 2014; Lawn et al., 2014; World Health Organization, 2014a, b; Nikolaou et al., 2015; Suzuki et al., 2015; Halberstadt, 2017; Marchi et al., 2019). In order to improve buccal absorption, 25I-NBOMe may be complexed with cyclodextrin. Other less common routes of administration include oral, rectal, vaginal, intravenous or intramuscular injection, and smoking (EMCDDA, 2014; World Health Organization, 2014a, b). Doses and duration of action depend on the route of administration (see Table 1).

According to the limited information available from user websites and clinical case reports, NBOMes have been sold as a ‘legal’ alternative to LSD (“legal LSD”) or as LSD due to the very potent psychedelic activity (Bersani et al., 2014; EMCDDA, 2014; World Health Organization, 2014a, b; UNODC, 2017). 25I-NBOMe was found in blotters seized in China sold as LSD (Zhang et al., 2018). A recent report from Columbia documents identification of 25I-NBOMe in 21 out of 70 blotters marked as LSD; the drug was combined in the same blotter with MDMA, 25I-NBOMe amine, and/or 25H-NBOMe (Mendoza-Valencia et al., 2019). NBOMes (25I-, 25C-, 25B-, and 25H-) were also found in tablets sold as Ecstasy (Chia et al., 2019). Thus, users may accidentally ingest NBOMe as counterfeited LSD or MDMA. According to information given on drug fora, users may find that LSD has a slight metallic taste or no taste at all, while 25I-NBOMe will have a bitter taste. The two substances can also be tested using a black light/UV source: LSD will glow whereas 25I-NBOMe will not (Zamnesia Blog, 2016).

Similar to other NPSs, users may combine NBOMes with various psychoactive substances: psychedelics (e.g., 2C compounds, mescaline, psilocybin and LSD), empathogens, psychostimulants, and/or depressants, including alcohol and marijuana, and medicines, both intentionally and unintentionally (Halberstadt, 2017; Madsen et al., 2017; Marchi et al., 2019). Importantly, the use of serotoninergic drugs, e.g., selective serotonin re-uptake inhibitors (SSRIs) or MAO-A inhibitors and/or substances known to increase extracellular serotonin levels may increase the risk of developing serotonergic toxicity, the symptoms of which include tachycardia, hypertension, hyperthermia, muscle rigidity, and convulsions (Volpi-Abadie et al., 2013).

## Effects Related to Use of NBOMes

NBOMes are used for recreational purposes and psychedelic/hallucinogenic experiences. Subjective ‘positive’ effects reported by users resemble those of other psychedysleptics and include euphoria, mild stimulation, mood lift, feelings of love and empathy, change in perception, ego softening, insight, brightened and vibrant colors, enhanced appreciation of music, strong closed/open eye visuals, enhanced tactile sensation, mental/physical stimulation, increase in associative and creative thinking, erotic, sexual thoughts and sensations, and life-changing spiritual experiences (Zuba et al., 2013; EMCDDA, 2014; World Health Organization, 2014a, b; Erowid.org). “*Wow! Visuals are crazy, and the music is intense*–*waves of 3D objects have taken over my living room and everything looks beautiful! […] Amazing party drug! I don’t feel very stimulated even though this is a psychedelic stimulant? But euphoria I feel quite allot, and this is a really social drug, although on high doses it’s a bit hard to have a real conversation.”* “*I literally FEEL the beauty of the universe in its infinite complexity. My perception of myself is erased. There is no longer a ‘me”’.* “*I am most certainly in a profound psychedelic headspace” (i.e., I intuitively understand the universe, society, etc. It’s much more of a pure psychedelic than its non-benzyl substituted cousin 2C-I while still retaining some of the entactogen qualities.”* (Erowid.org).

NBOMes produce an array of adverse effects (Hill et al., 2013; Rose et al., 2013; Bersani et al., 2014; EMCDDA, 2014; Forrester, 2014; Grautoff and Kähler, 2014; Lawn et al., 2014; Stellpflug et al., 2014; Suzuki et al., 2014; Tang et al., 2014; World Health Organization, 2014a, b; Hieger et al., 2015; Nikolaou et al., 2015; Poklis et al., 2015b; Srisuma et al., 2015; Wood et al., 2015; Gee et al., 2016; Kristofic et al., 2016; Hermanns-Clausen et al., 2017; Humston et al., 2017; Madsen et al., 2017; Rajotte et al., 2017; Schetz et al., 2017; Wiergowski et al., 2017; Zygowiec et al., 2017; Marchi et al., 2019; Erowid.org); for comprehensive reviews see Suzuki et al., 2015; Halberstadt, 2017).

### Psychoactive

Severe agitation, agitated delirium, intensive unpleasant hallucinations, aggression that sometimes progresses to violent and self-destructive behavior, paranoia, suicidal attempts, psychosis with delusions, dysphoria, irritability, fear, and panic attacks.

### Neurological

Hyperthermia, convulsions, clonus, motor incoordination, mouth numbing and impaired speech, insomnia, blurred vision, and leucoencephalopathy.

### Cognitive

Loss of location and time, confusion, short-term memory deficits, cognitive impairment, mental fatigue, altered mental state, loosening of association, and disorganized thoughts.

### Cardiovascular

Tachycardia, hypertension, cardiac arrest, and vasoconstriction leading to ischemia.

### Miscellaneous

Nausea, vomiting, sweating/chills, diaphoresis, tachypnea, respiratory and metabolic acidosis, leukocytosis, hyperglycemia, hyperkalemia, muscle rigidity, and compartment syndrome.

In severe cases, the use of NBOMes can led to comas, disseminated intravascular coagulation, liver failure, heart failure, pulmonary edema, cardiopulmonary arrest, rhabdomyolysis [a case of massive rhabdomyolysis with serum kinase creatinine concentration over 500,000 U/I was reported after ingestion of a 25I-NBOMe containing party pill named “Alice in Wonderland” (Schetz et al., 2017)], acute kidney failure, and multiorgan failure.

Srisuma et al. (2015) analyzed 148 cases of intoxication with NBOMe drugs and 193 with 2C compounds reported to the National Poison Data System in the United States from 1st September 2012 to 30th September 2014. They reported higher numbers of hallucinations/delusions, single-episode seizures, and benzodiazepine administration in NBOMe exposures (40.5, 8.8, and 50.0%, respectively) than those of 2C exposures (25.4, 3.1, and 32.6%, respectively).

In general, the features of NBOMe toxicity are also induced by other psychedelics. The main difference is an intensity and frequency of severe intoxication symptoms. The incidence of seizures is higher with NBOMes compared with other psychedysleptics, whereas muscle spasms, hyperreflexia, and tremors are rarely noted in cases of intoxication with NBOMes. The progression from rhabdomyolysis to metabolic acidosis, anuria, and acute renal failure is a common complication of severe NBOMe toxicity, but this is reported less frequently in cases of intoxication with other drugs.

By analogy to other NPSs, except for opioids and benzodiazepines, at present there are no specific antidotes for NBOMes, and all treatments used are symptomatic. Clinical management of acute toxicity resulting from the use of NBOMe compounds consists of monitoring, including fluids, electrolytes, acid-base balance, and supportive treatment: mechanical ventilation and intravenous administration of fluids; benzodiazepines (e.g., midazolam and lorazepam) given intravenously are used for sedation, to treat aggression, tremors, and convulsions; an infusion of catecholamines (noradrenaline, dopamine) to overcome sinus bradycardia; antiarrhythmic drugs (e.g., cardioselective β-blockers, amiodarone) to treat supraventricular tachyarrhythmia; and antipyretics/mechanical cooling in cases of hyperthermia. Gross hematuria and anuria require continuous venovenus hemodialysis (CVVHD), while oliguria demands CVVHD with citrate calcium. Patients with hematological disturbances require transfusion(s) of blood preparations (frozen plasma, frozen erythrocytes, or platelet concentrate). Severely aggressive patients may require antipsychotic drugs in addition to benzodiazepines (Hill et al., 2013; Rose et al., 2013; Forrester, 2014; Stellpflug et al., 2014; Hieger et al., 2015; Gee et al., 2016; Humston et al., 2017; Schetz et al., 2017; Wiergowski et al., 2017). Some emergency interventions are specifically intended to treat rhabdomyolysis, which may lead to severe complications, particularly acute kidney injury/failure and metabolic acidosis. They include discontinuation of further skeletal muscle damage by infusion of muscle relaxants (midazolam, rocuronium), early and aggressive fluid administration with a goal of maintaining an urinary flow of 200–300 mL/h, as well as urine alkalization to prevent myoglobin precipitation in a renal tract and management of hyperkalemia and hypocalcemia (Tang et al., 2014; Cervellin et al., 2017; Schetz et al., 2017)

Table 2 presents clinical fatality cases due to intoxication with NBOMes.

**TABLE 2:** Fatal cases related to use of NBOMe compounds.

Gender/Age	Case data	Toxicological findings	References
**25I-NBOMe**			
M/21	At a rave party, the decedent took two ‘hits of acid’ and smoked marijuana. On the way home with his friend, he was driving a car. He started hallucinating, damaged the interior of the car and caused a car accident. The man died before the arrival of the emergency services. Autopsy: Ecchymoses of the forehead and face, erythema around earlobes, hemorrhages in the conjunctivae.	25I-NBOMe and Δ^9^-THC metabolite were detected in heart blood and urine.	Walterscheid et al., 2014
F/15	A woman ingested a clear, unknown liquid at the party. On the way home she became irresponsible and was taken to a hospital. On arrival she had asystole and 39.9 °C temperature (rectal). She died approx. 18 min after arrival to the hospital. Autopsy: Hemorrhages of the conjunctivae. Endotracheal tube and oropharyn were filled by a white foam. Many abrasions and contusions over the shoulders, left hip and right buttock.	25I-NBOMe and Δ^9^-THC metabolite detected in heart blood and urine.	Walterscheid et al., 2014
F/23	Following insufflation of a white powder she believed to be “synthetic LSD”, a woman began acting strangely and uttering random words; she became aggressive, then vomited, had convulsions, collapsed and died. Autopsy: Multiple bruises and abrasions on most of the body. Some dried apparent vomitus was presented on her face. Congested lungs and mild pulmonary edema.	Aortic blood: 25I-NBOMe, 28 μg/L; 25H-NBOMe, 1 μg/L; 25C-NBOMe, 0.7 μg/L; Methamphetamine, 0.39 μg/L; Δ^9^-THC 3.4 μg/L	Kueppers and Cooke, 2015
M/19	Following ingestion of a blotter paper with “acid”, a man began behaving strangely and appeared paranoid. He went for a walk with his friends, but abruptly walked away from them. The decedent started to hallucinate, had delusions and finally jumped/fell from his balcony. Autopsy: Multiple blunt impact, injuries of the heart, aorta, liver and spleen. The skull displayed a large number of fractures. No non-traumatic abnormalities or lesions were identified.	25I-NBOMe (direct analysis): peripheral blood, 405 pg/mL; heart blood, 410 pg/mL; urine, 2860 pg/mL; vitreous humor, 99 pg/mL; bile,12.1 ng/g; brain, 2.78 ng/g; liver, 5.64 ng/g.	Poklis et al., 2014b
M/16	The decedent was at the party with his friends where he used a drug that was spotted on a blotter paper. In the morning his friend found a body and a number of pieces of glass in the floor. Autopsy: Abrasions on the chest, arms, neck, knees and shins. Presence of many red contusions. Mild cerebral edema, severe and diffuse pulmonary edema present within all five pulmonary lobes (right > left). The bronchial tree contained frothy edematous fluid.	25I-NBOMe: heart blood, 19.8 ng/mL; urine, qualitative identification.	Shanks et al., 2015
M/15	A teenager was at a party where he ingested 25I-NBOMe and mushrooms. Also he was drinking an unknown fluid. About an hour later, he started to vomit and convulse and finally lost consciousness. Three days later he died at the hospital from multi-system organ failure following unsuccessful cardiopulmonary resuscitation.	Serum: Δ^9^-THC, 4.1 ng/mL; 11-nor-9-carboxy-THC, 83 ng/mL. Blood: 25I-NBOMe, 0.76 ng/mL. Urine: 25C-NBOMe, 25H-NBOMe, 25I-NBOMe and psilocin, qualitative identification.	Lowe et al., 2015
F/17	A girl presented in status epilepticus shortly after ingesting an unknown substance on a blotter paper at a music concert. She then acutely developed hyperthermia, metabolic acidosis, rhabdomyolysis, elevated transaminases, acute kidney injury, hypokalemia and hypocalcemia. Later on she developed irreversible cerebral edema and died after 7 days.	Whole blood: 25I-NBOMe, 0.25 ng/mL; lithium 0.34 mmol/L.	Umemura et al., 1988
**25B-NBOMe**			
M/18	After ingesting ‘NBOMe’ in the form of a blotter paper, the decedent started to behave destructively, collapsed and became unresponsive. The man was transported to the local hospital where he died. Autopsy: Trauma like lacerations, abrasions and contusions. severe and diffuse pulmonary edema present within all five pulmonary lobes, gastric content in the bronchi.	Heart blood: 25B-NBOMe, 1.59 ng/mL; Δ^9^-THC, 2.4 ng/mL; 11-nor-9-carboxy-THC, 17.1 ng/mL; caffeine, qualitative identification. Urine: 25B-NBOMe, qualitative identification; 11-nor-9-carboxy-THC, 361 ng/mL.	Shanks et al., 2015
M/20	After ingestion of a recreational drug called ‘Blue Magic Master’, the decedent became agitated and experienced convulsions. He developed serotonin syndrome the next day and died two days later.	Plasma: 25B-NBOME, 3.15 ng/mL after admission; 0.45 ng/mL and 0.16 ng/mL, at six and twelve h post-admission, respectively.	Shintani-Ishida et al., 2018
M/teenager	The decedent was at the party where alcoholic beverages were present, as well as various drugs: marijuana, hashish, cocaine and ‘hallucinogenic blotters’. On the way back home, he reportedly reiterated “I want to kill myself” before jumping into the waterway. Autopsy: Cause of death: drowning.	Heart blood: 25H-NBOMe, 0.13 ng/mL; 25C-NBOMe, 1.43 ng/mL. Central blood: Δ^9^-THC, 9.9 ng/mL; 11-nor-9-carboxy-THC, 8.5 ng/mL.	Morini et al., 2017
M/23-24	Three men had a party in a flat on the fifth floor. One of them jumped out of a window and was found dead.	Blood: 25B-NBOMe, 661 ng/mL; 4-CMC, 0.8 ng/mL	Wiergowski et al., 2017
	The other two were aggressive and very agitated. They were shouting at each other, speaking illogically, screaming incomprehensibly and moving anxiously in the flat.		
	One of them experienced strong convulsions, heavy breathing and salivation; he died at the hospital. The second man survived. He had a bag with him with the description: “25B NBOMe 1 GR” and “4 CMC 1 GR”.	Blood: 25B-NBOMe, 66.5 ng/mL; 4-CMC, 2.14 ng/mL	
M/19	After consumption of LSD, a man with no known past medical history, started to behave oddly and uncontrollably. He was sent to rest in a bedroom, where was found unresponsive one h later. The deceased died in a hospital after unsuccessful cardiopulmonary resuscitation. Cause of death: 25B-NBOMe toxicity.	25B-NBOMe: blood 10 ng/mL; bile and stomach content - present	Chan et al., 2019
**25C-NBOMe**			
M/22	The deceased had a short history of alcohol and drug use. After sniffing 25C-NBOMe, he started running and pulling down the curtains in his house, experiencing hallucinations, clenched jaw and convulsions. At the hospital, the following symptoms were observed: hyperthermia with a core temperature of 40 °C, pulse of 140 bpm, diffuse bleeding from all mucosa, respiratory and	25C-NBOMe: peripheral blood 0.6 μg/kg, urine 2.93 μg/kg, liver 0.82 μg/kg, vitreous humor 0.33 μg/kg, gastric content 0.32 μg total. Amphetamine: peripheral blood 470 μg/kg.	Andreasen et al., 2015
	metabolic acidosis, rhabdomyolysis, high lactic acid, anuria and hyperkalemia. He died approximately 12 h after ingestion due to the multiorgan failure. Autopsy: Free fluid in thorax and abdomen, mucosal hemorrhage. Bruises and abrasions.	Δ^9^-THC: peripheral blood 1.5 μg/kg	

## Biotransformation

In recent years, biotransformation studies have been carried out for many NBOMe compounds. The metabolites have mainly been identified via *in vitro* study with microsomes and polled human hepatocytes or by the analysis of mouse or rat urine or authentic human samples of blood and urine collected from drug users. An accumulating body of data clearly indicates that NBOMes undergo extensive biotransformation that results in the production of numerous metabolites. For example, Caspar et al. (2015, 2017, 2018c, 2018d) list more than 60 metabolites for each of the analogs, 25B-NBOMe, 25C-NBOMe, and 25I-NBOMe, as well as 36 phase I and 33 phase II metabolites for 4-EA-NBOMe, 17 phase I and 21 phase II metabolites for 3,4-DMA-NBOMe, and 19 phase I and 14 phase II metabolites for 4-MMA-NBOMe. The calculated intrinsic clearance values for 25I-NBOMe and 25I-NBOH were found to be 70.1 and 118.7 mL/min/kg, respectively (Nielsen et al., 2017).

**FIGURE 2:**
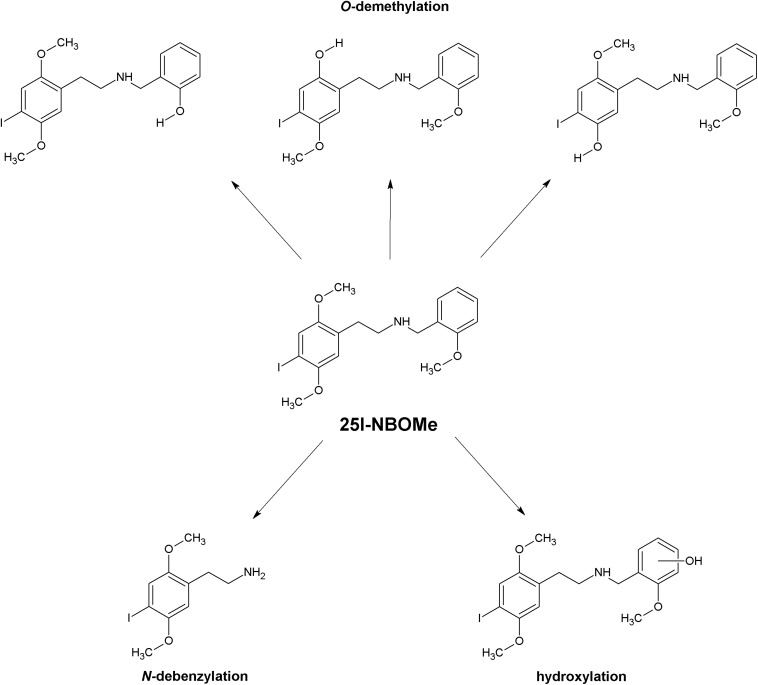
Main phase I metabolic pathways of 25I-NBOMe.

The reported biotransformation steps include oxidative deamination, oxidative *N*-dealkylation also in combination with hydroxylation, oxidative *O*-demethylation possibly combined with hydroxylation, oxidation of secondary alcohols, mono- and dihydroxylation, oxidation of primary alcohols, and carboxylation of primary alcohols (Figure 2). In the case of 25N-NBOMe, reduction of the aromatic nitro group and *N*-acetylation of the primary aromatic amine have also been reported. The dominant phase I biotransformation was *O*-demethylation, followed by *O*-di-demethylation and hydroxylation; accordingly, the most abundant metabolites were the *O*-demethylated and hydroxylated forms. The major cytochrome P450 isoenzymes involved in the metabolism of NBOMes were identified as CYP1A2, CYP3A4, CYP2B6, CYP2C9, CYP2C19, and CYP2D6 (Caspar et al., 2015, 2018b,c,d; Nielsen et al., 2017; Richter et al., 2019). Phase I metabolites subsequently undergo glucuronidation and sulfation (Caspar et al., 2015; Leth-Petersen et al., 2016; Temporal et al., 2017; Wohlfarth et al., 2017; Richter et al., 2019; Seo H. et al., 2019).

Forensic casework samples have also identified demethyl metabolites of 25C-NBOMe (Soh and Elliott, 2014; Andreasen et al., 2015). Poklis et al. (2015a) analyzed urine samples from two patients intoxicated with 25I-NBOMe. One sample contained 25I-NBOMe together with 15 metabolites, while the other contained no parent 25I-NBOMe; it was found to contain three *O*-demethyl metabolites. Seven 25I-NBOMe metabolites were detected in the urine of a severely intoxicated man: two demethyl-25I-NBOMe, one demethyl-hydroxy-25I-NBOMe, one hydroxy-25I-NBOMe, one di-demethyl-25I-NBOMe, one demethyl-25I-NBOMe glucuronide, and one hydroxy-25I-NBOMe glucuronide (Richeval et al., 2017). The presence of 25H-NBOMe in biological samples of people who used both 25B-NBOMe and 25I-NBOMe (Soh and Elliott, 2014; Stellpflug et al., 2014) suggests an alternative route of NBOMes biotransformation, i.e., removal of the halogen atom. However, it is also possible that 25H-NBOMe is not formed during metabolic processes in the body but, more likely, is already present in the consumed product as a contaminant. Assuming that the halogenation step performed during synthesis in a clandestine laboratory was incomplete and the unreacted material was not adequately removed by purification, it appears likely that 25H-NBOMe could be present in the final drug product.

The fact that NBOMe compounds undergo extensive first-pass metabolism by the liver (Leth-Petersen et al., 2016; Halberstadt, 2017) fits well with data demonstrating very fast clearance of parent compounds from plasma. This makes the signals of the parent compounds approximately 100-fold lower than those of the most abundant metabolites (Stellpflug et al., 2014; Leth-Petersen et al., 2016). The significantly greater intensity of glucuronated metabolites when compared to the parent compounds in plasma make them prime candidates to be used as markers for NBOMe intoxication (Leth-Petersen et al., 2016).

An important issue worth pointing out is the fact that metabolites can also be responsible for the toxic effects of NBOMes (Leth-Petersen et al., 2016). Two other groups of active formed compounds, which are also sold on the drug market, include 2C phenethylamines or NBOH derivatives (Pasin et al., 2015; Nisbet et al., 2019). For all investigated NBOMes, the corresponding 2,5-dimethoxyphenethylamine (2C-X) metabolite formed during *N*-demethoxybenzylation was detected; however, they were mostly seen at low levels (Temporal et al., 2017; Grafinger et al., 2018).

## Detection and Identification of NBOMes and Their Metabolites in Biological Materials

Due to the high receptor affinities of NBOMes and functional activities as full agonists, only very low doses, often in the range of 50–1000 μg, are needed to induce psychoactive effects, and, as a consequence, the resulting biological fluid concentrations tend to be very low, ranging from about 0.1 ng/mL to several ng/mL in blood and up to several dozen ng/mL in urine. Hence, only sensitive and specific analytical methods can be used for the detection, identification, and determination of NBOMes in biological materials (Kyriakou et al., 2015). Even in post-mortem cases, the reported blood concentrations of NBOMe compounds also tend to be low and are often below 0.5 ng/mL (Anilanmert et al., 2018). Although the compounds are generally present at higher concentrations in urine than in blood, detection methods should be targeted to the metabolites rather than the parent compounds.

The analysis of NBOMes can be a challenging task, even for laboratories equipped with sensitive modern methods, and popular immunochemical tests are not effective. Common analytical methods used in laboratories, such as gas chromatography coupled with mass spectrometry (GC-MS) or high-performance liquid chromatography with diode array detection (HPLC-DAD) without derivatization of the sample, are also inadequate for identifying NBOMe compounds due to insufficient sensitivity. Analytical methods must have low limits of detection (LOD); therefore, the most common techniques of detection of NBOMes in biological fluids are those implementing tandem mass spectrometry (MS-MS). Both high-performance liquid chromatography (HPLC or LC) and ultra-performance liquid chromatography (UPLC) coupled to either MS-MS or high resolution time-of-flight spectrometry (TOF-MS) are preferred. This latter technique allows for accurate determination of molecular and fragmentation ions, which in turn makes it possible to elucidate the chemical structure of compounds and consequently unambiguously identify not only the parent substance but also many metabolites. For the isolation of NBOMes from blood (as well as serum or plasma) and urine both liquid-liquid extraction (LLE) and solid phase extraction (SPE) can be used. Sometimes, a simple precipitation or just a dilution of a sample is sufficient.

Although many methods have been developed for the detection of NBOMe analogs in biological materials, both in metabolic studies and authentic forensic sample analyses, only a few screening methods covering more than one or two NBOMe compounds have been published. Caspar et al. (2018a) developed a method for the identification and determination of 21 low-dosed psychedelics and opioids, including 25B-NBOMe, 25C-NBOMe, 25E-NBOMe, 25I-NBOMe, and 25H-NBOMe, in blood plasma. A diethyl ether:ethyl acetate mixture was applied for a two-step extraction. Analyses were carried out using LC high resolution (HR) MS (orbitrap analyzer) with alternating HR full scan (HRFS) MS and “All-ions fragmentation” (AIF) MS. The approach allowed the detection of these analytes down to concentrations of 0.1 ng/mL.

Pasin et al. (2015) developed and validated an analytical method for the detection and quantification of 37 new designer drugs, including 25B-NBOMe, 25C-NBOMe, 25H-NBOMe, and 25I-NBOMe in the whole blood. Salting-out-assisted LLE with acetonitrile was performed to isolate compounds, followed by LC with an analysis combined with a quadrupole time-of-flight mass spectrometer (Q-TOF-MS). The method required only 100 μL of blood, but the limits of detection for NBOMe compounds was relatively high at 5 ng/mL. Temporal et al. (2017) describe the analysis of the same set of NBOMes with their major metabolites in blood and urine samples by UPLC-Q-TOF system; authentic samples underwent LLE before analysis using an n-butyl chloride:ethyl acetate mixture.

Poklis et al. (2014a) described the use of the LC-MS-MS method for the identification and quantification of nine NBOMe derivatives (25H-NBOMe, 2CC-NBOMe, 25I-NBF, 25D-NBOMe, 25B-NBOMe, 2CT-NBOMe, 25I-NBMD, 25G-NBOMe, and 25I-NBOMe) in human urine. The method used dilution of urine samples and extraction by Clean Screen FASt^TM^ SPE columns to reduce the amount of matrix. A Q-Trap apparatus was used in multiple reaction monitoring (MRM) acquisition mode, which allowed the compounds to be detected at a level of 0.1 ng/mL. SPE was also used to identify and quantify five different 25-NBOMes (25B-NBOMe, 25C-NBOMe, 25D-NBOMe, 25H-NBOMe, and 25I-NBOMe) in blood and urine. The applied LC–MS-MS (Q-Trap) system allowed LOD to be obtained at a level of 0.05 ng/mL (Morini et al., 2017).

The UPLC-MS-MS (Q-Trap) system was used for the simultaneous quantification of six NBOMe analogs (25B-NBOMe, 25C-NBOMe, 25D-NBOMe, 25H-NBOMe, 25I-NBOMe, and 25T2-NBOMe) in the whole blood, plasma, and urine. The SPE was performed with the use of UCT Clean Screen DAU mixed mode columns. The method, characterized by LODs as low as 0.005–0.01 ng/mL, offered sufficient sensitivity to detect any of these compounds following use (Johnson et al., 2014).

Caspar et al. (2017) analyzed 25B-NBOMe and 25C-NBOMe along with their metabolites in human and rat urine. Depending on the experiment, urine was either incubated with a mixture of glucuronidase/arylsulfatase and then extracted with the use an HCX SPE columns or just precipitated with acetonitrile. The obtained samples were analyzed on the LC-HR-MS/MS system. In subsequent studies, nanoLC-HRMS/MS and UHPLC-HRMS/MS systems were applied for the detection and identification of metabolites of 3,4-DMA-NBOMe and 4-MMA-NBOMe (Caspar et al., 2018c). Urine samples were analyzed directly after dilution. Mass spectrometers were operated in positive ionization mode using full scan (FS) data and a subsequent data-dependent acquisition (DDA) mode. Both applied systems were comparable, but nanoLC allowed much lower eluent consumption: flow rate of 0.7 μL/min for nanoLC compared to 500–800 μL/min for UHPLC. Wohlfarth et al. (2017) also employed the simple dilution of urine to analyze samples with or without a prior hydrolysis step (with ß-glucuronidase/arylsulfatase mixture). 25C-NBOMe and 25I-NBOMe, and their metabolites, were detected and identified by LC-QTOF-MS in DDA mode (Wohlfarth et al., 2017).

Yu et al. (2019) proposed a new method of NBOMes identification. The fragmentation patterns of nine NBOMe derivatives (25H-NBOMe, 25B-NBOMe, 25E-NBOMe, 25N-NBOMe, 25C-NBOH, 25I-NBOH, 25B-NBF, 25C-NBF, and 25I-NBF) were analyzed using LC-QTOF-MS and an approach known as molecular networking, one that organizes MS-MS data by mining the MS-MS fragmentation similarity. The resulting MS-MS spectral data was used to establish a molecular networking map for different NBOMes, as these compounds generally showed similar product ion spectral patterns. The map was applied to spiked urine samples, confirming that it can be used for the rapid detection and identification of unknown NBOMes.

In addition to body fluids, other biological materials have also been analyzed for NBOMe compounds, including post-mortem tissues. Tissue homogenates were subjected to SPE using UCT mixed mode silica-based columns. LC–MS-MS analyses were performed on a Q-Trap apparatus (Kristofic et al., 2016). A validated method for the detection of 32 NPS, including 25C-NBOMe, 25B-NBOMe, and 25T4-NBOMe, in oral fluid has also been presented in which samples were prepared using a simple protein precipitation in acetonitrile and analyzed using the UHPLC-MS-MS system. All analytes were found to have a LOD at 1 ng/mL (Williams et al., 2017). Ameline et al. (2017) presented an analysis of hair samples (9.5 cm) collected in an acute poisoning case. The hairs were collected 6.5 months after a drug consumption and were analyzed by UPLC-MS-MS working in MRM mode. As a result of the analyses, the presence of 25I-NBOMe was demonstrated in two of five 2 cm hair segments at concentrations of 1.0 pg/mg and 4.9 pg/mg.

GC-MS is used less frequently but it can be a great tool in screening analyses when used in combination with a derivatization step. A validated GC-MS method for the quantification of 23 NPSs, including 25B-NBOME, 25C-NBOME, 25D-NBOMe, 25E-NBOME, 25H-NBOME, 25I-NBOME, Mescaline-NBOME, and 25P-NBOME in blood and urine samples have been presented. Sample preparation was carried out using SPE followed by derivatization with pentafluoropropionic anhydride (PFPA). The LODs for NBOMes were in the range of 0.2–0.3 ng/mL in urine and 0.3–0.4 ng/mL in blood. It should be emphasized that the proposed method can be used for detection of NBOMes in acute fatalities by laboratories that do not have access to an LC–MS-MS (Nisbet et al., 2019).

## Concluding Remarks

In recent years, NBOMe derivatives, a specific set of psychedelic phenylalkylamines, have been encountered on the drugs of abuse market. These compounds are used in very low doses (in the range between 50 and 1000 μg) due to their high pharmacological activity. NBOMe drugs are highly toxic and their intake has been associated with severe adverse reactions including deaths. The analysis of NBOMes and their metabolites is a challenging task; only sensitive and specific analytical methods can be used for their detection, identification, and determination in biological materials.

## Author Contributions

JZ: literature search, writing the sections “Abstract,Introduction, Methods, Pharmacology of NBOMes, Toxicity in vitro, Available forms of products, Pattern of use, Effects related to use of NBOMes, and Concluding remarks.” MK: preparation of tables and co-writing the section “Effects related to use of NBOMes.” PA: writing the sections “Detection and identification of NBOMes and their metabolites in biological materials, Biotransformation”; preparation of figures, and literature search.

## Conflict of Interest

The authors declare that the research was conducted in the absence of any commercial or financial relationships that could be construed as a potential conflict of interest.
